# Eupatilin alleviates airway remodeling via regulating phenotype plasticity of airway smooth muscle cells

**DOI:** 10.1042/BSR20191445

**Published:** 2020-01-17

**Authors:** Yanqi Li, Rong Ren, Lijun Wang, Kan Peng

**Affiliations:** 1 Department of Respiratory, Xi’an Children’s Hospital, Xi’an 710003, Shaanxi Province, P.R. China; 2 Department of Otolaryngological, Hospital of Northwest Polytechnical University, Xi’an 710072, Shaanxi Province, P.R. China; 3 Department of Joint Surgery, Xi’an Hong Hui Hospital, Xi’an Jiaotong University Health Science Center, Xi’an 710054, Shaanxi Province, P.R. China

**Keywords:** airway remodeling, Childhood asthma, eupatilin, nuclear factor-kappa B (NF-κB), phenotype plasticity, signal transducer and activator of transcription 3 (STAT3)

## Abstract

Childhood asthma is a common chronic airway disease, and its severe form remains a challenge. Eupatilin is a bioactive natural flavone that has been found to possess potential anti-asthma activity. However, the roles of eupatilin in asthma remain to be elucidated. In the present study, airway smooth muscle cells (ASMCs) were applied for the *in vitro* investigation since their phenotype plasticity make great contribution to airway remodeling during asthma pathogenesis. Our results showed that eupatilin suppressed the transforming growth factor β1 (TGF-β1)-induced proliferation and migration of ASMCs. Exposure of ASMCs to eupatilin increased the expressions of contractile markers smooth muscle α-actin (α-SMA) and myocardin, whereas expressions of extracellular matrix (ECM) proteins type I collagen (Coll I) and fibronectin were reduced. Furthermore, eupatilin treatment reversed the activation of nuclear factor-κ B (NF-κB), signal transducer and activator of transcription 3 (STAT3) and AKT pathways caused by TGF-β1 in ASMCs. These findings suggested that eupatilin might attenuate airway remodeling via regulating phenotype plasticity of ASMCs.

## Introduction

Childhood asthma is a common chronic airway disease with substantially increased prevalence over recent decades [1]. Asthma is a chronic inflammatory disease, which is characterized by inflammation, reversible airway obstruction, hyperresponsiveness, and airway remodeling [1]. In the pathophysiology of asthma, chronic inflammation may lead to structural changes in the airways, such as thickening of the epithelial basement membrane, deposition of extracellular matrix (ECM) in the subepithelial layer, hyperplasia and hypertrophy of airway smooth muscle cells (ASMCs), which are referred to as airway remodeling [2,3]. ASMCs are key cell type in the pathophysiology of airway remodeling because of their multifunctional properties and intrinsic plasticity in response to inflammatory cell products [4]. Several inflammatory mediators have been proven to play important roles in airway remodeling. The cytokine transforming growth factor β1 (TGF-β1) has been reported to stimulate the proliferation and migration of ASMCs, as well as induce ECM protein synthesis and profibrotic differentiation of ASMCs, thereby promoting airway remodeling [5].

Eupatilin, a bioactive flavone of Artemisia asiatica Nakai (Asteraceae), has been reported to have broad properties such as anti-cancer, antioxidant, and anti-inflammatory effects [6]. In addition, Kim et al. [7] reported that eupatilin has anti-allergic reactions in activated guinea pig lung mast cells, suggesting that eupatilin may be used for the treatment of inflammatory disorders associated with allergic diseases such as asthma. Jeon et al. [8] demonstrated that eupatilin inhibits the expression of eotaxin, which is a chemokine contributing to the pathogenesis of asthma. Infiltration of eosinophils into respiratory tissue induced by inflammatory chemotactic factors is a characteristic feature of allergic diseases [9]. Eupatilin has been found to suppress TNF-α-induced eosinophil migration [8]. These data indicate that eupatilin may possess anti-asthma activity. However, the roles of eupatilin in asthma remain unclear. Thus, we aimed to investigate the effects of eupatilin on phenotype plasticity of ASMCs and the possible mechanism.

## Materials and methods

### Cell culture and treatments

Human ASMCs line (American Type Culture Collection, ATCC, Manassas, VA, U.S.A.) were grown in complete Dulbecco’s modified Eagle’s medium (DMEM; HyClone, Logan, UT, U.S.A.) supplemented with 100 mg/ml streptomycin, 100 U/ml penicillin, and 10% FBS (HyClone). ASMCs were maintained in a humidified atmosphere of 5% CO_2_ at 37°C. For some experiment groups, ASMCs were induced by TGF-β1 (20 ng/ml) for 48 h.

### Cell cytotoxicity assay

To evaluate the cell cytotoxicity of eupatilin on ASMCs, ASMCs were incubated with series concentrations of eupatilin (0, 10, 20, 40, 80 μM) for 48 h. Then the cell viability was detected by MTT assay. MTT solution was added to the cells at a final concentration of 0.2 mg/ml, followed by incubation at 37°C for 4 h. Subsequently, 200 μl dimethylsulfoxide (DMSO) was added to the cells to dissolve the crystals. The OD value at 490 nm was measured using a microplate reader (Bio-Tek, Winooski, VT, U.S.A.).

### Cell proliferation assay

Cell proliferation of ASMCs was evaluated using the Cell Counting Kit-8 assay (CCK-8; Dojindo, Kumamoto, Japan) according to the manufacturer’s instructions. Briefly, ASMCs were seeded in 96-well plates at the density of 1 × 10^4^ cells/well. Cells were pretreated with various concentrations of eupatilin (10, 20, 40 μM) for 1 h, followed by incubation with TGF-β1 (20 ng/ml) for 48 h. Then, 10 μl of CCK-8 reagent was added to each well and incubated for 3 h. The OD value was detected at 450 nm using a microplate reader (Bio-Tek, Winooski, VT, U.S.A.).

### Transwell migration assay

Transwell assay was performed using Transwell® culture chambers (Corning Life Sciences, Corning, NY, U.S.A.). The lower chamber was filled with 500 μl DMEM containing 10% FBS with TGF-β1 (10 ng/ml). ASMCs (1 × 10^5^) resuspended in 500 μl serum-free DMEM were placed in the upper chamber. TGF-β1 (10 ng/ml) was added to the cells with or without the presence of eupatilin (10, 20, 40 μM), and incubated for 24 h. After removing the non-migrated cells on the upper side of the inserts using a cotton swab, the migrated cells on the lower side of the inserts were fixed using 95% methanol for 10 min and stained with 0.5% Crystal Violet for 30 min. The average cell number from five random fields was calculated.

### Quantitative real-time polymerase chain reaction

Total RNA of ASMCs was extracted by using TRIzol (Invitrogen, Carlsbad, CA, U.S.A.). The obtained RNA was subjected to MultiScribe Reverse Transcriptase (Applied Biosystems, Foster City, CA, U.S.A.) to synthesize cDNA according to the manufacturer’s instructions. Quantitative real-time polymerase chain reaction (qRT-PCR) was performed using SYBR Green I Kit (Roche Diagnostics, Mannheim, Germany) on an ABI 7500 sequence detector system (Applied Biosystems). Expressions of the target genes were normalized to GAPDH using the 2^−ΔΔ*C*_T_^ method. The specific primers used in the present study were as follows: smooth muscle α-actin (α-SMA), forward 5′-AGAG TTAC GAGT TGCC TGAT GG-3′ and reverse 5′-GATG CTGT TGTA GGTG GTTT CA-3′; myocardin, forward 5′-AGGT AACA CAGC CTCC ATCC TA-3′ and reverse 5′-TGGG TATC TTTG GGAC TTTT TG-3′; type I collagen (Coll I), forward 5′-GTCC TCCT GGTT CTCC TGGT-3′ and reverse 5′-GACC GTTG AGTC CGTC TTTG-3′; fibronectin, forward 5′-GAAG TCGC AAGG AAAC AAGC-3′ and reverse 5′-GTAG GTGA ACGG GAGG ACAC-3′; GAPDH, forward 5′-AGAA GGCT GGGG CTCA TTTG-3′ and reverse 5′-AGGG GCCA TCCA CAGT CTTC-3′.

### Western blot

Total protein was extracted from ASMCs using RIPA lysis buffer (Fermentas, Glen Burnie, MD, U.S.A.). After determination of protein concentration, equal amount of protein extracts was separated by 10–12% SDS/PAGE and transferred to polyvinylidene difluoride (PVDF) membranes (Millipore, Billerica, MA, U.S.A.). After incubation with blocking buffer, 5% non-fat milk, for 1 h, primary antibodies against α-SMA (1:500; Abcam, Cambridge, MA, U.S.A.), myocardin (1:500; Sigma–Aldrich, St. Louis, MO, U.S.A.), nuclear factor-κ B (NF-κB) p65 (1:500; Sigma–Aldrich), p-p65 (1:500; Abcam), signal transducer and activator of transcription 3 (STAT3; 1:500; Sigma–Aldrich), p-STAT3 (1:500; Sigma–Aldrich), p-AKT (1:1500; Abcam), AKT (1:2000; Abcam), and β-actin (1:500; Abcam) were added to the incubation system and incubated at 4°C overnight. Then the membranes were incubated with horseradish peroxidase-labeled secondary antibody (1:5000; Abcam) at 37°C for 1 h. The protein bands on the membranes were visualized using ECL reagent (ECL, Pierce, Rockford, IL, U.S.A.). The protein levels were analyzed by detecting the density using ImageJ Software (National Institutes of Health, NIH, Bethesda, MD, U.S.A.).

### ELISA

Spontaneous release of Coll I and fibronectin in ASMCs supernatant was determined by commercial ELISA kits (R&D Systems, Minneapolis, MN, U.S.A.) following the manufacturer’s instructions.

### Statistical analysis

All data were presented as mean ± standard deviation (SD), and statistical analysis was performed using SPSS version 23.0 software (SPSS Inc., Chicago, IL, U.S.A.). One-way analysis of variance (ANOVA) was used to compare the differences among multiple groups. Differences with a *P*-value <0.05 were considered statistically significant.

## Results

### Effect of eupatilin on cell proliferation and migration in TGF-β1-induced ASMCs

**Figure 1:**
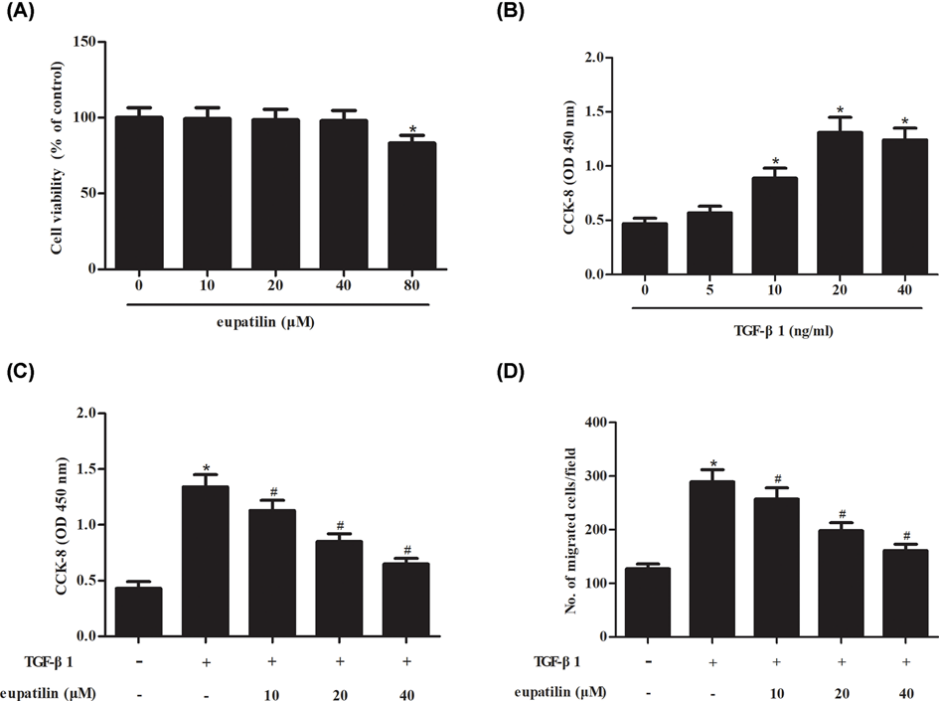
Eupatilin significantly inhibited TGF-β1-induced ASMCs proliferation and migration (**A**) Cell cytotoxicity of eupatilin on ASMCs. ASMCs were incubated with series concentrations of eupatilin (0, 10, 20, 40, 80 μM) for 48 h. MTT assay was performed to evaluate cell viability. (**B**) ASMCs were stimulated with different concentrations of TGF-β1 ranging from 0 to 40 ng/ml for 48 h, and cell proliferation was measured using CCK-8 assay. (**C**) ASMCs were pretreated with eupatilin (10, 20, or 40 μM) for 1 h, and then incubated with TGF-β1 (20 ng/ml) for 48 h. CCK-8 assay was performed to assess cell proliferation. (**D**) Cell migration of ASMCs. Transwell assay was conducted to evaluate cell migration. *n*=5. *P<0.05 vs. control cells; ^#^*P*<0.05 vs. TGF-β1-induced ASMCs.

In order to evaluate the cell cytotoxicity of eupatilin on ASMCs, ASMCs were incubated with series concentrations of eupatilin (0, 10, 20, 40, 80 μM) for 48 h. MTT assay proved that eupatilin did not exhibit cytotoxicity on ASMCs even at the concentration of 80 μM (Figure 1A). Then we selected the concentrations of 10, 20, and 40 μM for further experiments.

Next, to examine the effect of TGF-β1 on ASMCs proliferation, ASMCs were treated with different concentrations of TGF-β1 (0, 5, 10, 20, 40 ng/ml) for 48 h. The results showed that TGF-β1 dose-dependently stimulated ASMCs proliferation, and 20 ng/ml TGF-β1 was selected to induce ASMCs (Figure 1B). Then, ASMCs were pretreated with eupatilin (10, 20, or 40 μM) for 1 h, and then incubated with TGF-β1 (20 ng/ml) for 48 h. CCK-8 assay showed that TGF-β1 significantly induced cell proliferation, while the induction was attenuated by eupatilin pretreatment (Figure 1C). Transwell assay indicated that cell migration was elevated after TGF-β1 induction, but dose-dependently reduced by eupatilin pretreatment (Figure 1D).

### Effect of eupatilin on contractile phenotypic markers in TGF-β1-induced ASMCs

**Figure 2:**
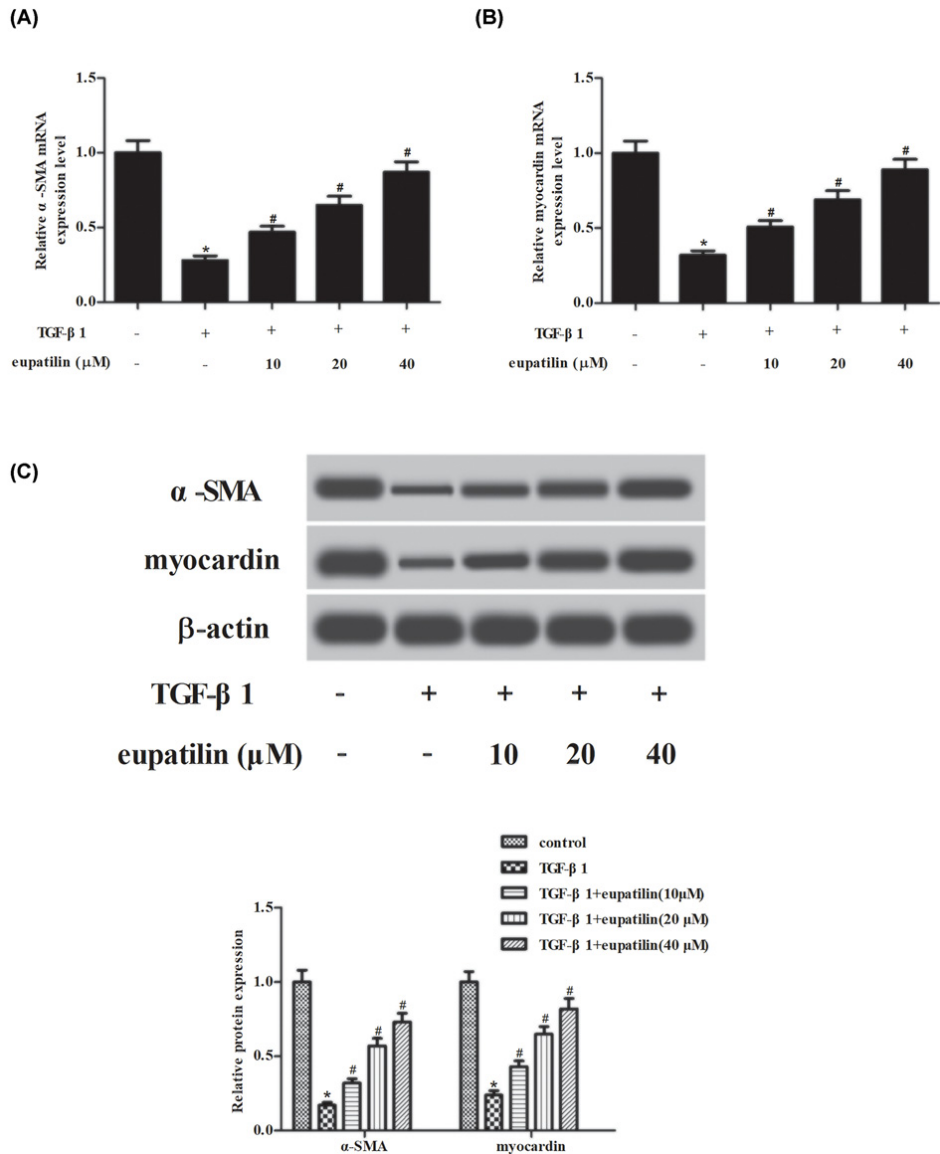
Eupatilin reversed the TGF-β1-induced inhibition of the expression of contractile phenotypic markers in ASMCs ASMCs were pretreated with eupatilin (10, 20, or 40 μM) for 1 h, and then incubated with TGF-β1 (20 ng/ml) for 48 h. The mRNA and protein levels of contractile phenotypic markers including α-SMA and myocardin were measured using RT-PCR (**A,B**) and Western blot analysis (**C**), respectively. *n*=4. *P<0.05 vs. control cells; ^#^*P*<0.05 vs. TGF-β1-induced ASMCs.

Expressions of contractile phenotypic markers including α-SMA and myocardin were measured using RT-PCR and Western blot analysis, respectively. As shown in Figure 2A,B, treatment with TGF-β1 decreased the mRNA levels of α-SMA and myocardin in ASMCs. In addition, the protein levels of α-SMA and myocardin were also reduced after TGF-β1 treatment (Figure 2C). However, the inhibitory effects of TGF-β1 on α-SMA and myocardin were mitigated by eupatilin pretreatment in a dose-dependent manner.

### Effect of eupatilin on ECM proteins in TGF-β1-induced ASMCs

**Figure 3:**
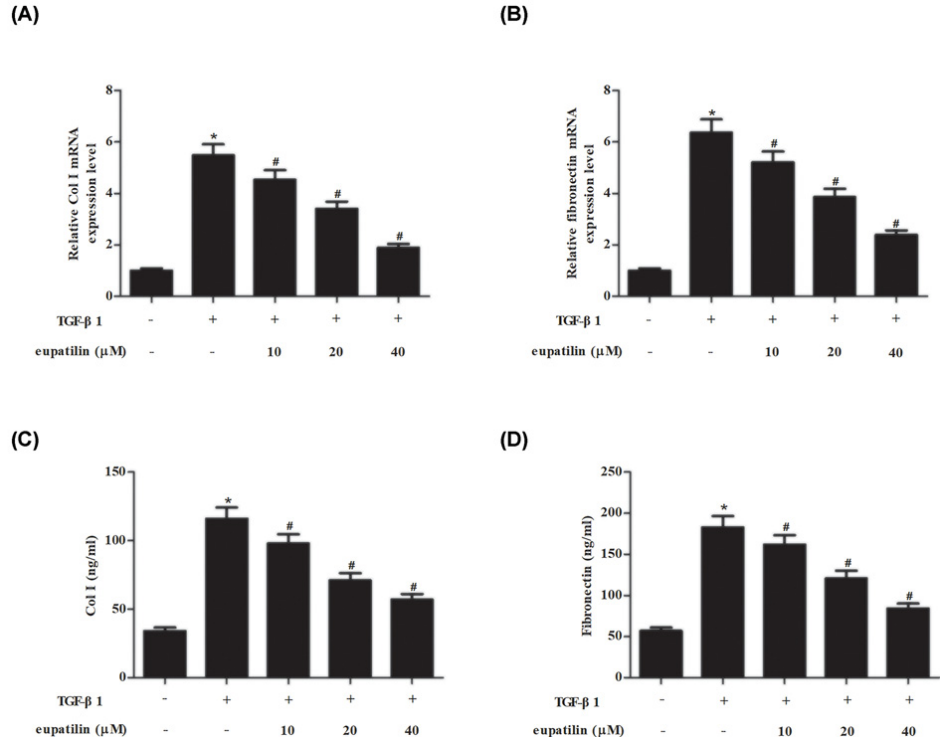
Eupatilin suppressed Coll I and fibronectin production in TGF-β1-stimulated ASMCs ASMCs were pretreated with eupatilin (10, 20, or 40 μM) for 1 h, followed by incubation with TGF-β1 (20 ng/ml) for 48 h. (**A**–**D**) The mRNA levels of Col I and fibronectin in ASMCs and the secretion in cell supernatants were determined using RT-PCR and ELISA, respectively. *n*=4. *P<0.05 vs. control cells; ^#^*P*<0.05 vs. TGF-β1-induced ASMCs.

The mRNA levels of ECM proteins including Coll I and fibronectin in ASMCs were determined using RT-PCR. As shown in Figure 3A,B, TGF-β1 caused significant increase in mRNA levels of Coll I and fibronectin, while eupatilin noticeably suppressed the induction. Then the production of Coll I and fibronectin in supernatant was assessed using ELISA. We found that eupatilin clearly inhibited the TGF-β1-induced secretion of Coll I and fibronectin in ASMCs (Figure 3C,D).

### Effect of eupatilin on NF-κB, STAT3 and AKT pathways in TGF-β1-induced ASMCs

**Figure 4:**
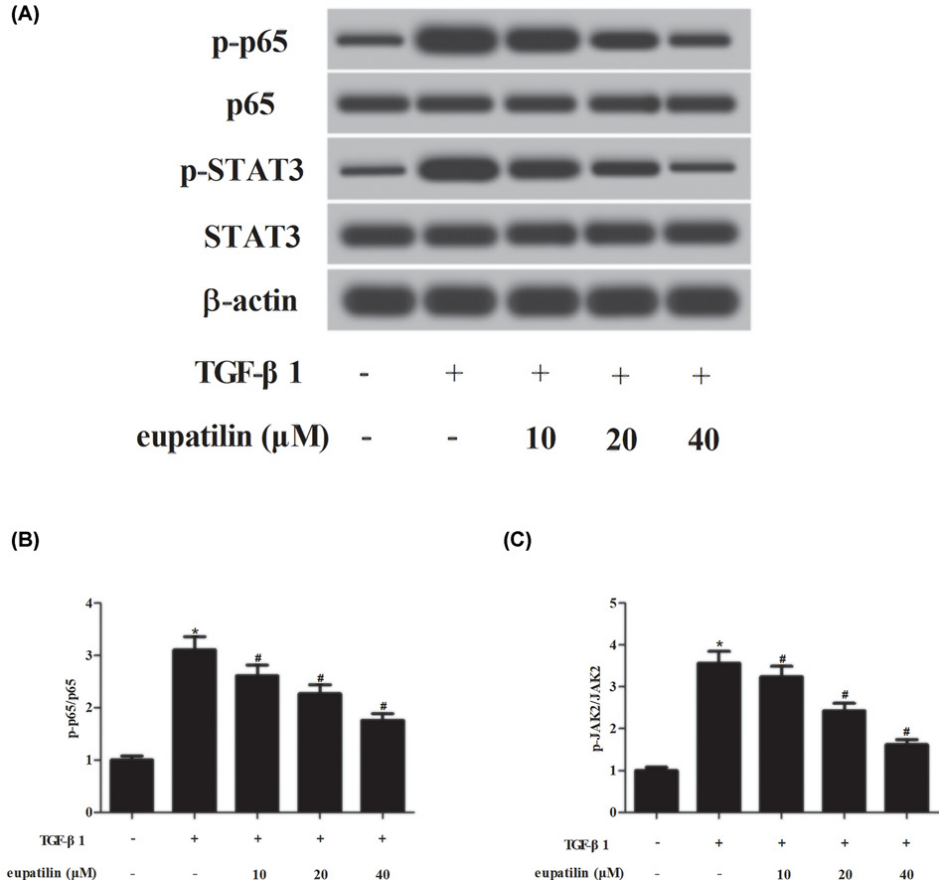
Eupatilin prevented the activation of NF-κB and STAT3 pathways in TGF-β1-stimulated ASMCs (**A**) After TGF-β1 stimulation for 2 h with or without the pretreatment of eupatilin (10, 20, or 40 μM), the expressions of p65, p-p65, STAT3, and p-STAT3 were measured using Western blot. (**B**) The ratio of p-p65/p65. (**C**) The ratio of p-STAT3/STAT3. *n*=3. *P<0.05 vs. control cells; ^#^*P*<0.05 vs. TGF-β1-induced ASMCs.

**Figure 5:**
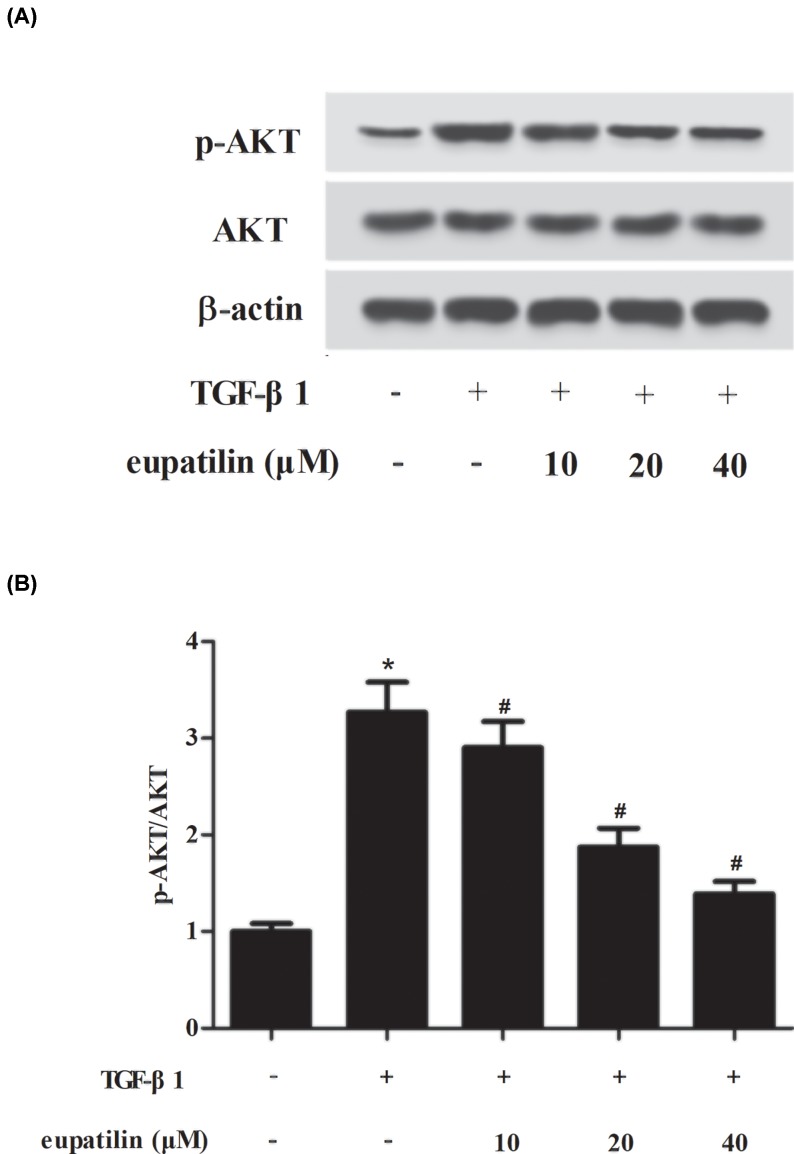
Eupatilin prevented the activation of AKT pathway in TGF-β1-stimulated ASMCs (**A**) After TGF-β1 stimulation for 30 min with or without the pretreatment of eupatilin (10, 20, or 40 μM), the expressions of p-AKT and AKT were measured using Western blot. (**B**) The ratio of p-AKT/AKT. *n*=3. *P<0.05 vs. control cells; ^#^*P*<0.05 vs. TGF-β1-induced ASMCs.

To identify the signaling pathways that mediate the effects of eupatilin, we determined the alternations of NF-κB and STAT3 pathways by detecting the expressions of p65, p-p65, STAT3, and p-STAT3. The results of Western blot revealed that TGF-β1 significantly elevated the expression levels of p-p65 and p-STAT3, indicating that TGF-β1 resulted in activation of NF-κB and STAT3 pathways (Figure 4). However, the activation of NF-κB and STAT3 pathways caused by TGF-β1 was reversed by eupatilin treatment. Furthermore, we observed that eupatilin inhibited TGF-β1-induced AKT pathway activation in ASMCs (Figure 5).

## Discussion

ASM plays crucial roles in airway remodeling during asthma pathogenesis, in part due to its phenotype plasticity [10,11]. ASMCs have the ability to change the degree of a variety of functions, including proliferation, migration, contractility, and secretion of inflammatory mediators [10]. During airway remodeling, ASM proliferation contributes toward increased ASM mass, which tends to increase airway narrowing and airflow obstruction [10]. Cell migration could be in part responsible for the pathogenesis of ASM hyperplasia and remodeling in asthma [10]. It has been proven that multiple stimuli contribute to airway remodeling represented by ASM proliferation or migration. ASMCs are usually for the *in vitro* studies of airway wall remodeling and phenotype plasticity in asthma [11]. Fang et al. [12] addressed that TGF-β1 induces ASMCs proliferation and migration. Chen et al. [13] demonstrated that triptolide functions as an inhibitor of asthma airway remodeling, which is evidenced by the inhibition of TGF-β1 induced ASMCs proliferation and migration. In the present study, we found that eupatilin suppressed cell proliferation and migration in TGF-β1-induced ASMCs, suggesting that eupatilin might have the ability to change the phenotype plasticity.

It is now well established that ASMCs are the major contractile elements and have central roles in determining airway structure and function [11]. In response to stimuli, ASMCs exhibit reversible switching between contractile and proliferative phenotypes, thereby driving remodeling [14]. This phenotype switching is also referred to as phenotypic plasticity [14]. The proliferating ASMCs have an increased proliferative capacity, more mitotically active, and express lesser amounts of contractile proteins such as smooth muscle myosin heavy chain (SM-MHC), α-SMA, calponin, myocardin, and desmin [15]. The phenotype modulation may be governed by a variety of growth factors present in the asthmatic airway, including TGF-β1. Our results showed that eupatilin reversed the TGF-β1-induced inhibition of the expression of contractile phenotypic markers in ASMCs. ASM is now recognized to be a source of ECM proteins that drive structural changes. Additionally, the ECM is not only a dynamic structure [16]. Previous studies have shown that ECM proteins, in particular Coll I and fibronectin, can alter non-asthmatic derived ASM cells toward a proliferative phenotype [4,17]. Moreover, composition of the ECM proteins can store a number of pro- and anti-inflammatory cytokines and growth factors, which can be released to modulate ASM proliferative and synthetic capacity, thus creating a complex network regulating the extent of airway remodeling [1,18]. The current study revealed that TGF-β1 caused significant increase in expressions of Coll I and fibronectin, while eupatilin noticeably suppressed the induction.

It has been previously investigated that various signaling pathways are involved in regulating phenotypic plasticity of ASMCs, such as the toll-like receptors (TLRs), NF-κB, mitogen-activated protein kinase (MAPK), Janus-activated kinase (JAK)/STAT [19–21]. Among these signaling pathways, NF-κB is an important participant in a broad spectrum of inflammatory networks [22]. Higher level of activated NF-κB is observed in asthma, various strategies targeting NF-κB signaling have been considered for asthma treatment [22]. STAT3 is an important transcription factor that has been found to be implicated in airway inflammation and remodeling in asthma [23]. Jeon et al. [8] reported that eupatilin inhibits NF-κB signaling in bronchial epithelial cells, leading to inhibition of eosinophil migration. Moreover, eupatilin inhibits angiogenesis in gastric cancer cells by blocking STAT3-mediated vascular endothelial growth factor (VEGF) expression [24]. Jung et al. [25] reported that eupatilin suppressed the activation of NF-κB, as well as suppressed the phosphorylation of Akt in TNF-α-stimulated BEAS-2B cells. Therefore, we investigated whether eupatilin could affect NF-κB, STAT3, AKT signaling pathways in TGF-β1-induced ASMCs. In accordance with previous studies, herein, our results showed that eupatilin inhibited the activation of NF-κB, STAT3, and AKT signaling pathways caused by TGF-β1 induction. Recently, Fei et al. [26] showed that eupatilin inhibited inflammatory response through the TLR4/MyD88 pathway in erythrocyte lysis stimulation-induced mouse microglia BV2. Whether eupatilin can attenuate the inflammatory response in asthma will require further studies.

In the present study, we evaluated the roles of eupatilin in ASMCs phenotypic modulation in response to TGF-β1 stimulation. We present evidence that eupatilin suppressed cell proliferation, migration, ECM accumulation, while elevated the expressions of contractile phenotypic markers in TGF-β1-induced ASMCs. The effects of eupatilin might be mediated by the inhibition of NF-κB, STAT3, and AKT signaling pathways.

## Abbreviations

ASMC: airway smooth muscle cell
CCK-8: cell counting kit-8
Coll I: type I collagen
DMEM: Dulbecco’s modified Eagle’s medium
ECM: extracellular matrix
NF-κB: nuclear factor-κ B
STAT3: signal transducer and activator of transcription 3
TGF-β1: transforming growth factor β1
TLR: toll-like receptor
α-SMA: smooth muscle α-actin
